# Ileal obstruction caused by transmural endometriosis in a patient with simultaneous C. difficile colitis and Influenza AH1N1. Case report

**DOI:** 10.1016/j.ijscr.2020.10.039

**Published:** 2020-10-12

**Authors:** Adriana Santos-Manzur, David Ricardo Valdez-Bocanegra, María Cristina Ornelas-Flores, Janet Pineda-Díaz, Enrique Stoopen-Margain

**Affiliations:** a Department of Surgery, ABC Medical Center, Mexico; b Department of Surgical and Molecular Pathology, ABC Medical Center, Mexico

**Keywords:** Ileal obstruction, Transmural endometriosis, Bowel resection, Anastomosis, Case report

## Abstract

**Introduction:**

Small bowel obstruction secondary to endometriosis is extremely rare. This etiology is reported in 0.10% of cases and diagnosed incidentally.

**Case presentation:**

32-year old female, critically ill patient was diagnosed with small bowel obstruction. After medical stabilization of multiple comorbidities, diagnostic laparoscopy was done and converted to open right ileocolectomy with ileo-transverse anastomosis. Definite diagnosis was ileal obstruction caused by transmural endometriosis. The patient's recovery was uneventful. At one-year follow-up, she remains asymptomatic.

**Discussion:**

We present the uncommon case of ileal obstruction due to transmural endometriosis that was treated surgically. We also present current literature review focusing on diagnostic and treatment methods of this rare disease.

**Conclusion:**

Endometriosis should be included in the differential diagnosis of small bowel obstruction. The treatment of choice is bowel resection.

## Highlights

• Bowel obstruction or intestinal occlusion caused by endometriosis is very rare.
• Endometriotic nodules induce luminal stenosis and ileal obstruction.
• Transmural endometriosis infiltrates the four layers of the intestinal wall.
• Diagnosis is established through pathological and immunohistochemical analysis.
• The treatment of choice is bowel resection via laparoscopy or laparotomy.

## 1 Introduction

Small bowel obstruction secondary to endometriosis is extremely rare. This etiology is reported in 0.10% of cases and diagnosed incidentally. Using the Surgical Case Report (SCARE) Guidelines [1], we present a case of a patient with ileal obstruction caused by transmural endometriosis with simultaneous C. difficile colitis and Influenza AH1N1. We also present current literature review focusing on diagnostic and treatment methods of ileal obstruction due to endometriosis.

## 2 Case report

A 32-year-old nulliparous woman presented into Emergency Room with colicky abdominal pain that lasted 48 h. Additionally, she had diarrhea, abdominal distension, nausea, vomiting, dry cough, nasal congestion, headache, and fever. She was taking antibiotics for H. pylori gastritis. Her medical history included endometriosis. She was taking oral contraceptives intermittently. She had right oophorectomy at age 22 due to endometrioma, and right salpingectomy at age 25 due to ectopic pregnancy. In-vitro fertilization and embryo transfer failed at age 29.

**Fig. 1:**
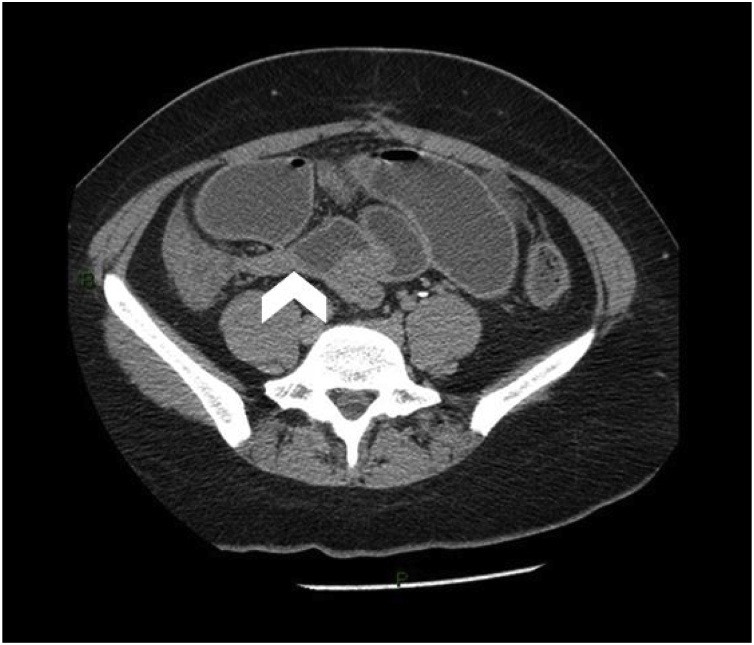
CT with IV contrast. Transition point at 5 cm from the ileocecal valve (white arrowhead).

On physical examination: BP 80/60 mmHg. HR 120 bpm. RR 22 bpm. Temp. 39 °C. Her abdomen was diffusely distended, absent bowel sounds, generalised tenderness, without rebound. Vaginal examination was unremarkable. Rectal exam showed liquid stool. Leucocytes 18,000/μL. PaO_2_/FiO_2_ ratio 370. Abdominal CT scan with IV contrast showed small bowel diameter of 5 cm with air-fluid levels and transition point at 5 cm from the ileocecal valve (Fig. 1). PCR was positive for C. difficile as well as Influenza AH1N1.

Patient was admitted in the Intensive Care Unit. She was kept NPO. A nasogastric tube was placed, and 2 L of enteric fluid were drained. She was started on Vancomycin 500 mg QID and Oseltamivir 75 mg BID by nasogastric tube, as well as Tigecycline 50 mg BID IV. The organ failure resolved within 48 h.

**Fig. 2:**
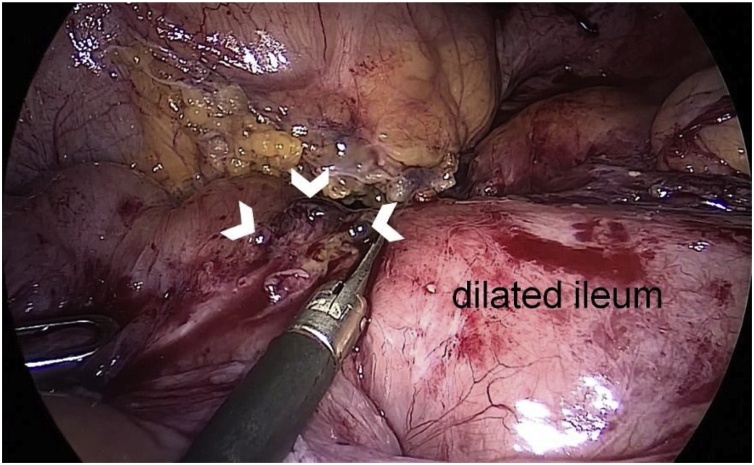
Intraoperative findings. Ileal obstruction induced by many blue-black endometriotic nodules (white arrowheads).

**Fig. 3:**
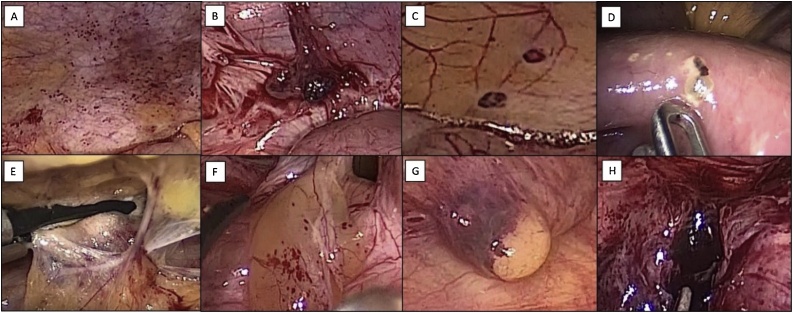
Laparoscopic findings. Peritoneal endometriosis: typical and atypical lesions. **A.** Red petechial lesions. **B.** Blue-black nodules. **C.** Red flame-like lesions. **D.** Intestinal implants and white lesions. **E.** Adhesions and scarring. **F.** Glandular excrescence and peritoneal defects. **G.** Subperitoneal nodule. **H.** Old blood collections.

On her second hospital day, laparoscopic exploration was performed. Small bowel was diffusely dilated, and extensive intra-abdominal and pelvic adhesions were seen. The point of obstruction was at the terminal ileum next to the ileocecal valve. That segment of bowel was severely stenosed. Ileal obstruction was induced by many blue-black nodules (Fig. 2). On the surface of the parietal and visceral peritoneum, many lesions were visualized and biopsied (Fig. 3).

Due to the complexity of the case, it was converted to midline laparotomy since the exposure was not adequate to perform the surgery safely. Extensive lysis of adhesions was done. At this point, right ileocolectomy was performed. Using a 60 mm stapler, ileo-transverse anastomosis was done. No complications were reported.

Patient's recovery was uneventful. She was started on total parenteral nutrition (TPN) on postoperative day (POD) 0. On POD 1, she had bowel sounds and slowly recovered her bowel function. On POD 4, she was stared on liquid diet. Her diet was slowly progressed and the TPN weaned. By POD 8, she was tolerating soft diet, having bowel function, and no signs of active infection. She was discharged home.

The macroscopic pathological examination consisted of a 20 cm long ileum segment and a 6 cm cecum and colon segment. The external surface was light brown, with congestive areas. The luminal surface had hematic material and showed a variable diameter secondary to stenotic areas, with purplish areas of hemorrhagic appearance. Cross sections showed small dilated cystic areas in relation to fibrosis and mural bleeding.

**Fig. 4:**
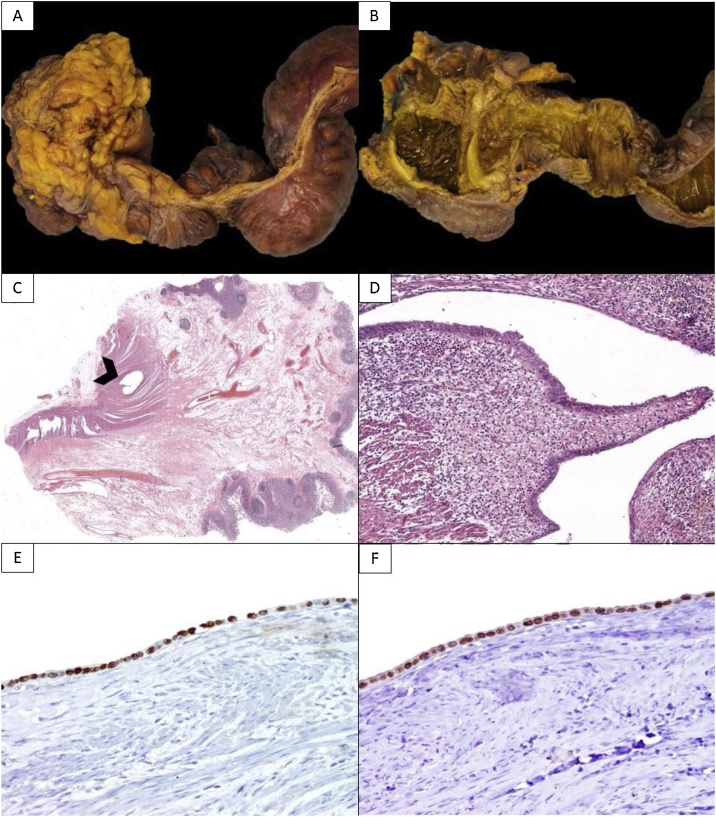
Pathological examination and immunohistochemical analysis. **A.** Gross external surface with congestive areas and diameter variability. **B.** Luminal surface with stenosis and hemorrhagic patches. **C.** Panoramic view of colonic wall with mucosal layer without alterations, numerous prominent lymphoid follicles with germinal centers in the submucosa, dilated and congestive vessels in lamina propria, and serosal fibrosis that surrounds glandular structures (black arrowhead) (H&E, 4x). **D.** High-power view of the glandular structures. They are irregular, lined by columnar epithelial cells, and surrounded by endometrial stroma, smooth muscle fibers and some lymphocytes, as well as hemosiderin-laden macrophages (right lower corner) (H&E, 20x). **E.** Positive immunostaining for Estrogen Receptor. **F.** Positive immunostaining for PAX8.

Microscopically, we found multiple foci along the enterocolonic wall of glandular and tubular structures, lined by epithelial cuboidal-columnar cells without atypia, surrounded by endometrial stroma with lymphoplasmacytic inflammatory infiltrate, focal hemorrhage, and hemosiderin-laden macrophages. There was important vascular proliferation as well as extensive subserosal fibrous bands. Immunostains for Estrogen Receptor and PAX8 were positive, which confirmed the endometrial nature of the lesions. The diagnosis of enterocolonic transmural endometriosis with serosal adhesions was established (Fig. 4).

Definite diagnosis was ileal obstruction secondary to transmural endometriosis. At one-year follow-up, patient remains asymptomatic. She has been referred to Gynecology for further treatment of endometriosis.

## 3 Discussion

Endometriosis is defined by ectopic endometrium [2]. 10% of the women in reproductive age have endometriosis [3]. Etiopathogenesis is multifactorial. This process has multiple interconnected factors both inherited and acquired [4]. The most common locations are ovaries, fallopian tubes, pouch of Douglas, and pelvic peritoneum [5]. The common clinical manifestations are dysmenorrhea, chronic pelvic pain, dyspareunia, and infertility [5].

From the patients with endometriosis, 10% have enterocolonic endometriosis [6]. From that subset of patients, 90% have rectosigmoid involvement [6], and only 10% of women have ileal endometriosis [3]. Most cases are asymptomatic [7]. Ileal endometriosis can be manifested with unspecific symptoms such as abdominal pain, distension, constipation, diarrhea, and changes in bowel habits [2]. Those symptoms can or not be related with the menstrual cycle [2]. Commonly those cases are misdiagnosed as irritable bowel syndrome or infectious enteritis [2].

Only 0.10% of women with ileal endometriosis develop ileal obstruction [8]. Physiopathogenesis implies both endometriotic nodule and ileal thickening that progressively lead to luminal stenosis and ileal obstruction [9]. Hypothalamus-hypophysis-ovarian-axis hormones influence cyclically the endometriotic nodule [9]. Estradiol induces its proliferation as well as its infiltration through ileal wall: from serosa towards mucosa. Progesterone produces its decidualization. Withdrawal of these hormones causes hemorrhage of endometriotic nodule and surrounding tissues. This process induces concentric wall thickening. Mural changes include hypertrophy and hyperplasia of lymphoid follicles, smooth muscle fibers, and neurons within submucous and myenteric plexus [9]. Fibroblastic hyperplasia produces extensive fibrosis and adhesions [9].

Ileal obstruction caused by endometriosis is diagnosed incidentally [10]. Luminal stenosis is usually located within 10 cm from the ileocecal valve [11]. Differential diagnoses of luminal stenosis are tuberculosis, Crohn’s disease, adenocarcinoma, lymphoma, gastrointestinal stromal tumor, and carcinoid tumor [2,12]. Histological diagnosis is straightforward by identification of endometriotic foci [5]. Diagnostic triad refers to endometrioid-type glands, endometrioid-type stroma, and hemosiderin-laden macrophages [9]. The diagnostic triad is usually present. Transmural endometriosis refers to the involvement of the serosa, the muscularis propria, the submucosa, and the mucosa of the intestinal wall, by endometriotic foci [9]. From the patients that present with obstruction, 10% have transmural endometriosis [13]. As was the case with our patient.

Ileal endometriosis is mostly identified in sections with hematoxylin and eosin staining. Immunohistochemical analysis is also useful. Immunostains most used are Estrogen Receptor (ER), Progesterone Receptor (PR), Pair-Box 8 (PAX8), and Cluster of Differentiation 10 (CD10) [14]. In our case, we used ER and PAX8. ER is diffuse nuclear positive in both endometriotic glandular-epithelial and stromal cells [14]. PAX8 is diffuse nuclear positive only in endometriotic glandular-epithelial cells [14]. ER and PAX8 have 100% of sensitivity and 100% of positive predictive value in detecting ileal endometriosis [14].

Bowel resection is the treatment of choice in patients with ileal obstruction due to endometriosis [6]. Surgical resection can be performed via laparoscopy or laparotomy. A multidisciplinary approach with Surgery, Gynecology, and Gastroenterology, is recommended [13].

Our case is unique. Ileal obstruction was secondary to transmural endometriosis. Additionally, our patient had C. difficile colitis and Influenza AH1N1 at the same time.

## 4 Conclusions

Ileal obstruction due to endometriosis is extremely rare and diagnosed incidentally. However, it should be contemplated as possibility in the differential diagnosis of women with small bowel obstruction. Definite diagnosis is established through pathological examination and immunohistochemical analysis. The treatment of choice is bowel resection.

## Conflicts of interest

None.

## Sources of funding

Sources of funding: This research did not receive any specific grant from funding agencies in the public, commercial, or not-for-profit sectors.

## Ethical approval

Ethical approval: Exception: Case report of interesting case encountered during normal medical practice.

## Consent

Written informed consent was obtained from the patient for publication of this case report and accompanying images. A copy of the written consent is available for review by the Editor-in-Chief of this journal on request.

## Author contribution

Adriana Santos-Manzur: treating physician, concept and design, data collection, data analysis, writing.

David Ricardo Valdez-Bocanegra: concept and design, data collection, data analysis, writing.

María Cristina Ornelas-Flores: data collection, data analysis.

Janet Pineda-Díaz: data collection, data analysis.

Enrique Stoopen-Margain: data collection, data analysis.

## Registration of research studies

Not applicable.

## Guarantor

Adriana Santos-Manzur.

## Provenance and peer review

Not commissioned, externally peer reviewed.
